# Preparation and Tensile Properties of Novel Porous Plates Made by Stainless Steel Wire Mesh and Powder Composites

**DOI:** 10.3390/ma14030677

**Published:** 2021-02-01

**Authors:** Shengcun Lin, Zhaoyao Zhou

**Affiliations:** 1 National Engineering Research Center of Near-Net-Shape Forming for Metallic Materials, School of Mechanical and Automotive Engineering, South China University of Technology, Guangzhou 510640, China; mesclin@mail.scut.edu.cn; 2 School of Automotive Engineering, Liuzhou Vocational & Technical College, Liuzhou 545005, China

**Keywords:** wire mesh and powder composite, tensile properties, rolling process, sintering parameters

## Abstract

Porous metal materials have important mechanical properties, and there are various manufacturing methods to produce them. In this paper, a porous, thin strip was fabricated by the composite rolling of stainless steel wire mesh and stainless steel powder. Then, a porous plate of stainless steel wire mesh and powder composite (SWMPC) was prepared by folding, pressing, and vacuum sintering the thin strip, and its structural characteristics and permeability were studied. The effects of the gap of the roller, gap of the powder box, number of layers by folding, and sintering parameters on the porosity and mechanical properties were also studied. The results indicated that the permeability increased with the increasing of porosity. Sintering parameters had a great influence on the mechanical properties. The larger the roll gap, the higher the porosity and the weaker the mechanical properties. As the gap of the powder box increased, the porosity decreased and the mechanical properties improved. The number of layers had no effect on the porosity. The first three stages of tensile curves of 10 and 15 layers were deformation stages and generally coincided, the time was short at the fracture stage. However, the mechanical properties got a raise when layers was 15.

## 1. Introduction

Metal porous materials are excellent multi-purpose engineering materials that can be used not only as functional materials but also as structural materials [1,2]. Metal porous materials also have a range of properties that compact metals do not have, such as energy absorption and buffering [3]. Because of their important physical and mechanical properties, metal porous materials are used widely in the engineering field [4]. The mechanical properties of metal porous materials depend on the original metal and the manufacturing processes. Porosity and pore structure also have great influence on the mechanical properties [5]; the tensile properties of metal porous materials are one of the indicators that cannot be ignored. Research on the tensile properties of porous materials has shown that the higher the porosity is, the lower will be the strength and stiffness [6,7]. The sintering temperature has an obvious effect on the tensile strength of the composite porous materials [7,8]. For example, Raghavendra et al. [6] studied the effects of different types of cell structures and porosities on mechanical properties. Irregular and regular cells and fully random porous structures were also studied through tensile and compression uniaxial tests, and the investigation showed that the strength and Young’s modulus decreased significantly with increasing porosity for quasi-static, cyclic compression, and tensile tests. The uniaxial tensile properties of the sintered multi-layer mesh porous plate were studied by Duan et al. [7] and they found that the higher the sintering temperature, the thicker were the coarser joints between the wires and the higher was the tensile strength. The sintering temperature decreased from 1330 to 1130 °C and the tensile strength decreased from 296 to 164 MPa. Porous FeAl intermetallics were prepared by Shu et al. [9] and scanning electron microscopy (SEM) experiments and uniaxial tensile tests were undertaken to understand the macroscopic mechanical properties and microscopic failure mechanisms. The results showed that the tensile curves of the porous FeAl with different porosities can be divided into four stages: elasticity, yielding, strengthening, and failure, without the necking phenomenon. The elastic modulus, ultimate strength, and elongation decreased with the increase of porosity and the elongation was much lower than 5%. Porous metal fiber/powder sintered composite plates were developed and tensile tests were conducted by Zou et al. [10] to study the tensile behavior of the composite plates. Results showed that the composite sheet successively experienced an elastic stage, hardening stage, and fracture stage under tension. With increased porosity of the composite sheet, the tensile strength of the composite sheet decreased. A uniaxial tensile test was carried out by Zhou et al. [11] to understand the effects of fiber length on the tensile properties of sintered porous metal fiber plates. The results showed that the increase of fiber length is helpful for enhancing the tensile strength of fibers under a given stress. The elongation of the sheet with medium length fibers of 15 mm exhibited the optimal performance, reaching about 13.5%.

There are many methods to prepare metal porous materials. A new method for the manufacturing of pre-determined open porosity Al foams was presented by Costanza et al. [12]. It is an alternative and cheap solution for tailoring metal foam to control the pore’s size and shape. A new type of sintered porous metal composite was prepared by mixing and sintering high porosity open-cell copper foam plates with spherical copper powder [8]. A novel technique for creating a uniformly-distributed space supporter was used to prepare 316l porous stainless steel foam. The new method was used to realize uniform pore distribution of the face-centered cubic packing by using spherical carbamide as the space supporter [13]. Porous Ti with the porosity of 50% and the average pore size of 200 μm was prepared by the powder metallurgy technique using polymethyl methacrylate as space holder [14]. A novel porous, metal fiber, sintered sheet with a three-dimensional network structure was produced via solid-state sintering of copper fibers [15]. A 24.0~35.5% porous titanium-tantalum composite was successfully prepared by sintering with 20 μm titanium powder and rice husk particles ranging in size between 250 μm and 600 μm [16]. However, there are only a few studies on the preparation of porous, thin strips by the composite rolling of stainless steel wire mesh and stainless steel powder, and the preparation and mechanical properties of a porous plate of wire mesh and powder composite created by folding, pressing, and vacuum sintering with a thin strip has not been studied so far.

In this paper, a porous, thin strip was fabricated by the composite rolling of stainless steel wire mesh (mesh count 400) and stainless steel powder (mesh count 200). Then, the porous plate of stainless steel wire mesh and powder composite (SWMPC) was prepared by folding, pressing, and vacuum sintering with the thin strip, and its structural characteristics were studied. The effects of rolling reduction, gap of the powder box, number of layers by folding, and sintering parameters on the porosity and tensile properties were also studied.

## 2. Materials and Methods

### 2.1. Preparations of Materials

**Figure 1:**
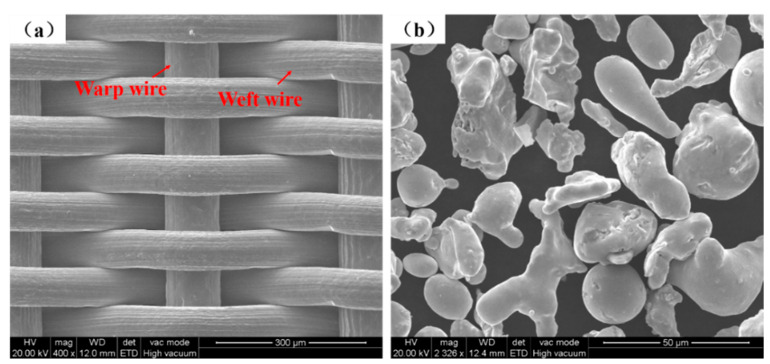
SEM images of the materials: (**a**) 304 stainless steel Dutch wire mesh; (**b**) 304 stainless steel powder.

According to the warp and weft of different weaving technologies, fabric structure can be classified into plain weave, twill weave, and satin weave [17]. Dutch wire mesh is improved on the basis of these three kinds of weaving technologies [18]. In the SEM image in Figure 1a, the weft wires are about 80 μm in diameter and clasp tightly to each other, while the warp wires are about 108 μm in diameter and run under the weft wires. This reasonable weaving method gives the Dutch wire a small size of mesh. Compared with other wire mesh with bigger apertures, such as plain wire mesh, the mechanical properties of the Dutch wire mesh are better [18]. In order to enhance the mechanical properties of the SWMPCs, 304 stainless steel Dutch wire mesh, with a mesh count of 400, as a reinforcement was combined with metal powder and rolled into a porous, thin strip. The powder was 304 stainless steel powder that was irregular and prepared by the water atomization method, and its mesh count was 200, shown in Figure 1b.

### 2.2. Design of Experiments

#### 2.2.1. Preparation of the Porous Plate of Stainless Steel Wire Mesh and Powder Composite

**Figure 2:**
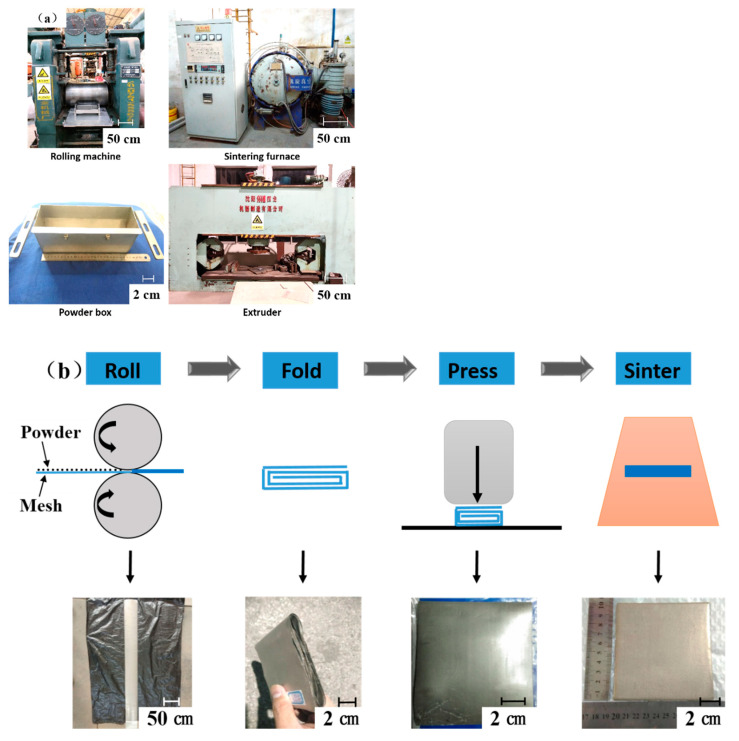
(**a**) The equipment of preparation; (**b**) The process of preparation.

As shown in Figure 2a, the two rollers of the rolling machine were arranged horizontally, one above the other, and the maximum rolling force was 240 tons. The samples of powder/mesh/plate was rolled by the machine. The gap between the two rollers was adjusted through control of the location of the roller that moves upwards. An auxiliary device, which included an aluminum plate and a powder box with a moving plate, was mounted on the rolling machine. Then, the porous, thin strip was prepared by rolling after the wire mesh and powder were laid. The process of preparation is shown in Figure 2b. In order to eliminate the gap between the layers, the porous, thin strip was pressed by the extruder, which had an extrusion pressure of 315 tons, after being folded into a certain number of layers. Finally, SWMPC was prepared using high temperature sintering and heat preservation for 2 h in a vacuum furnace. In order to avoid the oxidation of metal samples during the sintering process and ensure that the furnace stayed in a vacuum, the vacuum numerical panel in the furnace was maintained at a value lower than 3 Pa.

**Figure 3:**
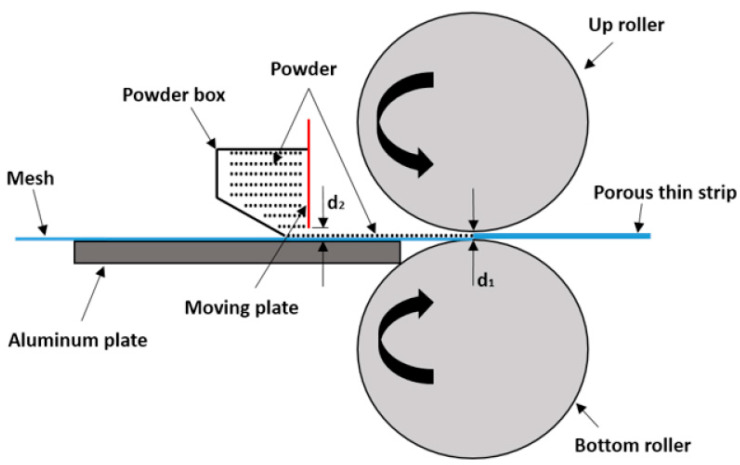
The rolling principle of the porous, thin strip.

As shown in Figure 3, the moving plate at the side of the roller can be moved up and down, and we defined the gap of the roller as d_1_ and the gap between the aluminum plate and the moving plate as d_2_. Both d_1_ and d_2_ were adjusted in advance of creating the samples. The wire mesh passed through the gap between aluminate and the moving plate and was driven forward by the rolling force between the two rollers. Metal powder was loaded into the powder box and flowed through the gap of powder box and was spread on the wire mesh evenly as the wire mesh was driven forward by the rollers. In that way, a thin layer of metal powder of certain thickness was formed. In the end, the metal powder was rolled along with the wire mesh into a thin strip.

#### 2.2.2. Experiment of Air Permeability

In most cases, gas is used as the test medium in the experiments of the permeability of porous materials [19], and the relative permeability coefficient of porous materials is regarded as the final characteristic [20]. There are many disadvantages in using liquid as the experimental fluid, because the fluid contains small solid particles that will change the permeability, also, some materials may be adsorbed into some liquid [21]. Compressed air is easy to obtain and clean, and so was selected for use in this paper.

**Figure 4:**
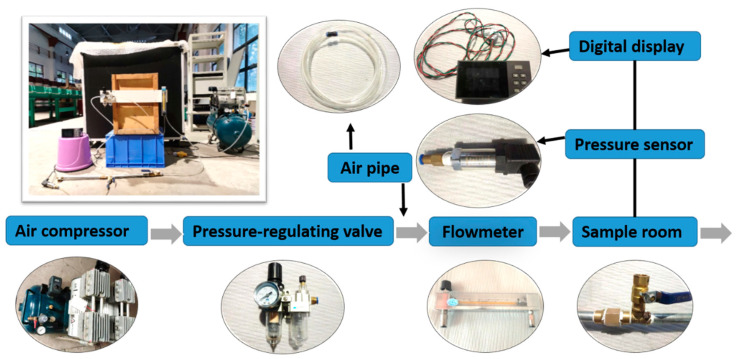
Experimental apparatus and principle of air permeability test.

As shown in Figure 4, a set of experimental equipment was designed for measuring the permeability of the porous plate accurately. First, a pressure-regulating valve controlled the gas input. Next, a flowmeter controlled the velocity of the gas. Finally, a pressure difference was produced when the gas flowed through the sample room. Then, the permeability of the porous material was calculated.

### 2.3. Characterization

#### 2.3.1. Microstructure Imaging

A scanning electron microscope (SEM, Quanta200, FEI, Eindhoven, Holland) was used to observe the surface and section morphology of the thin strip and porous plate, and the appearance of fractures in the tensile samples. The tensile test was performed with an electronic universal mechanical testing machine (NO: UTM5105, SUNS, Shenzhen, China).

#### 2.3.2. Porosity

The testing methods of porosity include microcosmic analysis, soaking method, floating method, and mass volume method, etc. [22,23,24,25]. There were no inclusions of different densities in the samples prepared with 304 stainless steel as the raw material in this paper. Moreover, the three-dimensional size could be measured easily because the shapes of the samples were all rectangular, so the mass volume method was used to measure the porosity using the following Equation:

(1) $$P\left(\%\right)=\left(1-\frac{M}{\rho V}\right)\times 100$$

where P is the average porosity of the sample, M is the mass of the sample (g), V is the volume of the sample (cm^3^), and ρ is the density of the original solid metal (g/cm^3^).

#### 2.3.3. Relative Permeability Coefficient

For convenience, relative permeability is used to characterize the permeability of porous materials in engineering. The simplified formula of Darcy [20] was used to calculate the permeability, which can be expressed as:

(2) $$K_{g}=\frac{Q}{A\Delta_{P}},$$

where $K_{g}$ is the relative permeability of the sample (m^3^/(h·kPa·m^2^)), Q is the fluid flow (m^3^/h), $\Delta_{P}$ is the pressure difference of gas flow through the sample (kPa), and A is the area of the sample that is perpendicular to the direction of the fluid.

## 3. Results and Discussion

### 3.1. Structural Characterization of the Porous Plate of Stainless Steel Wire Mesh and Powder Composite

**Figure 5:**
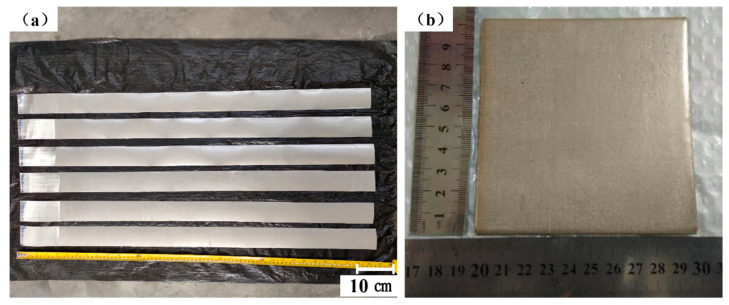
(**a**) The appearance of the porous, thin strip; (**b**) The appearance of the sample.

Look carefully at the thin strip shown in Figure 5a: The powder is firmly combined on the surface of the screen to form a thin powder layer. Since the diameter of the stainless steel powder (mesh count 200) is much larger than the mesh size of the 304 stainless steel Dutch wire mesh (mesh count 400), the back of the stainless steel sheet mesh is smooth and has no powder after rolling. The thin strip is soft and foldable without shedding powder.

As shown in Figure 5b, the sample was prepared by folding, pressing, and vacuum sintering with the thin strip. In ocular inspection, there are tiny pores with distinct pore characteristics on the surface of the sample, which is significantly different from what is seen on an ordinary stainless steel plate. The sample has a certain stiffness, which can be used for line cutting without deformation.

**Figure 6:**
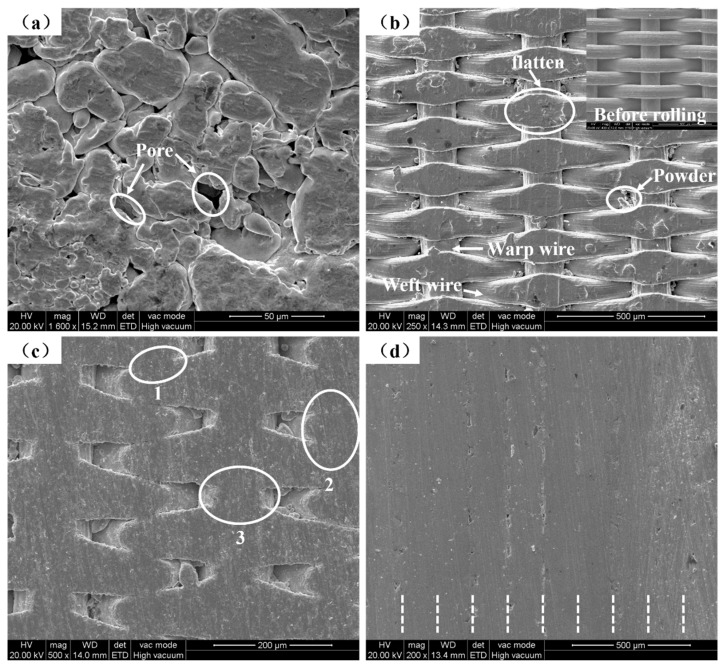
SEM images: (**a**) The porous, thin strip on the side with powder; (**b**) The porous, thin strip on the side without powder; (**c**) The surface of the sample; (**d**) Cross-section of the sample.

Powder covered the surface of the powder side of the porous, thin strip, and its micro-topographic image is shown in Figure 6a. Due to the rolling force, the powder was rolled into a flat shape and there were many tiny pores between powders. The microtopographic image of the surface without powder of the porous, thin strip is shown in Figure 6b. Due to rolling force, the warp wire and weft wire were rolled flat, especially at the position where the warp wire and weft wire overlap each other. Since the powder has a smaller mesh count than that of the wire mesh, some stainless steel powder oozed from the pores of the wire mesh.

The surface microtopography of the sample is shown in Figure 6c. There are sintering necks denoted by (1) and (2) in Figure 6c; a metallurgical bond is produced between weft wires that overlap closely to each other. Item (3) in Figure 6c shows a metallurgical bond produced at the position where the warp wire and weft wire overlap each other. Tiny pores are formed by the connection between the warp wire and weft wire, then constitute the surface pore distribution of the sample.

The sample was made by layering 10 porous, thin strips and using a sintering temperature of 1330 °C, and the cross-section morphology of the sample is shown in Figure 6d. Under the action of high temperature sintering, metallurgical bonds were produced between wire mesh and powder, powder and powder, and wire mesh and wire mesh, so the bonding between each layer was very tight and the interface combination was ideal. Each strip can be distinguished carefully, as illustrated by the short white dotted lines in Figure 6d.

Porous materials are important engineering materials [1,26,27], their permeability is also known as the relative permeability coefficient, which indicates the performance of porous materials and is an important index for characterizing the permeability of porous materials [28,29,30]. The relative permeability coefficient is the pressure difference caused by different flow rates of fluid flowing through porous materials, and it reflects that people pay more attention to the relationship between flow and pressure when using porous materials [31].

**Figure 7:**
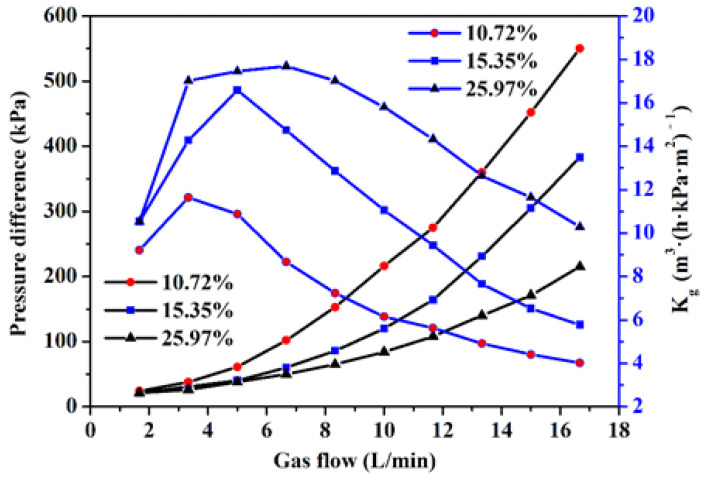
The curve of the permeability of the samples.

The porosity has a great influence on the permeability of porous materials. The greater the porosity, the more easily the fluid passes through, and the stronger is the permeability [32]. As shown in Figure 7, the SWMPCs have permeabilities that follow the law of most porous materials: With an increase of the fluid flow, the pressure difference with a porosity of 25.97% changed more slowly than that with a porosity of 10.72%, showing a stronger permeability. As shown in Table 1, the sample with a porosity of 10.72% showed the best permeability at a gas flow of 3.33 L·min^−1^, and the relative permeability coefficient was 11.64 m^3^·(h·kPa·m^2^)^−1^, while the sample with a porosity of 25.97% showed the best permeability at a gas flow of 6.67 L·min^−1^, and the relative permeability coefficient was 17.69 m^3^·(h·kPa·m^2^)^−1^, and the relative permeability coefficient of the samples increased by 51.98%. This new type of porous composite with permeability might be used for micro-porous filters [33,34,35] or novel porous restrictors [36,37].

### 3.2. Tensile Properties of the Porous Plate of Stainless Steel Wire Mesh and Powder Composite

**Figure 8:**
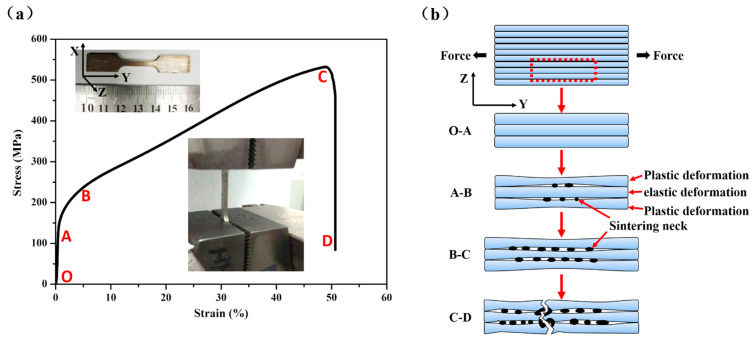
(**a**) The typical tensile stress-strain curve for the sample; (**b**) Fracture principle of the sample during tensile process.

Figure 8a shows the tensile stress-strain curves of the sample with porosity of 15.35% (No. S4). The technological parameters of the preparation process are as follows: d_1_ was 0 mm, d_2_ was 0.5 mm, the number of layers by folding was 10, and it was sintered at 1330 °C for 2 h. In Figure 8, the curve can be divided into four stages: elastic deformation, elastic and plastic deformation, plastic deformation, and fracture.

The elastic deformation stage (O–A): This is the initial stage, and the curve of this stage approximates to a straight line, indicating that the deformation at this stage satisfies Hooke’s law. The time of this stage is short, the elastic deformation, that cannot be observed, is less than 0.5%.

The elastic and plastic deformation stage (A–B): The curve of this stage is an arc and there is no obvious yield point, indicating that the elastic deformation and plastic deformation exist simultaneously at this stage. The reason why elastic and plastic deformations coexists is that they are determined by the properties of the material itself. The preparing technology resulted in uneven distribution of pore size and varying degrees of metallurgical bonding in the porous plate. Therefore, in the process of stretching, as shown in Figure 8b, due to the local stress concentration, some larger pores with poorer metallurgical quality deform first. The metallurgical bonding area that has larger pores and higher surrounding powder yield begins to turn to plastic deformation, as the yield at this area results in stress redistribution. Then, other areas that have smaller pores with good metallurgical quality begin yield and turn to plastic deformation, until all areas yield and turn to plastic deformation. Areas with large pores and poorer metallurgical bonding quality show plastic deformation, while in other areas elastic deformation occurs, which leads to the coexistence of elastic and plastic deformation.

The plastic deformation stage (B–C): The curve of this stage approximates to a straight line, showing that the process from the beginning of plastic deformation to yield point is gradual; the material has obvious tensile deformation behavior at this stage. As shown in Figure 8b, in the plastic deformation stage, the sample is obviously elongated. Similar to the plastic deformation stage of tight metal stretch, the deformation hardening of the sample increases with the increase of plastic deformation. The deformation hardening enhances the ability of the sample to resist further deformation and prevents the continuous plastic deformation, leading to the increase of stress. At this stage, the phenomenon of the stress increases is obvious, and the stress reaches the maximum until the deformation hardening reaches the limit of the sample.

The fracture stage (C–D): The curve of this stage is approximate to a vertical line, illustrating that the stage from yield point to complete failure is rapid. As the stress reaches the limit position, macroscopic cracks begin to appear on the sample, and the deformation is concentrated in the weak area of the sample. Then, the fracture occurs on the metallurgical bonding area, and then fractured phenomenon of other areas occur after stress redistribution. This phenomenon keeps happening and leads to a sharp drop in stress, until complete fracture of the sample occurs.

**Figure 9:**
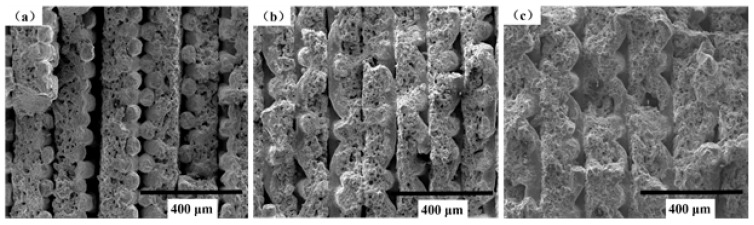
SEM images of fracture morphology after the tensile test. (**a**) Sintered at 1130 °C; (**b**) Sintered at 1230 °C; (**c**) Sintered at 1330 °C.

Three samples (No. S3, S7, S8) were fabricated with the following technological parameters: d_1_ was 0mm, d_2_ was 0.3 mm, the number of layers by folding was 10, and the sintering temperature was 1130 °C, 1230 °C, or 1330 °C, respectively. The porosities for the three samples were 18.27%, 18.3%, and 20%, respectively. As shown in Figure 9a, because of the sintering temperature was lower, the quality of combinations between sintering thin strips were poor, and the delamination was distinct between thin strips under the action of tensile force. There is no obvious sintered neck between the wire mesh and the powder, so the cylindrical shape of the wire mesh was clearly visible. As shown in Figure 9b, a fraction of the cylindrical shape of the wire mesh is still visible, but most of them have disappeared because of the sintering neck formed between the wire mesh and powder during high-temperature sintering. Metallurgical bonding occurred at the positions between the wire mesh and powder, wire mesh and wire mesh, and powder and powder in each and adjacent thin strips under the action of high-temperature sintering. The delamination between the thin strips occurred due to tensile force, but the phenomenon was not as serious as that shown in Figure 9a. This is because the sintering temperature increased by 100 °C, which makes the movement of internal atoms more intense, and tighter metallurgical bonding occurs at more areas during sintering. As shown in Figure 9c, the cylindrical shape of the wire mesh obviously disappeared. Even when the fracture occurred under the action of tensile force, the delamination between the thin strips that had close combination did not occur. This is because internal atoms are extremely displaced at the sintering temperature of 1330 °C. Metallurgical bonding obviously occurred at the positions between the wire mesh and powder, wire and wire, and powder and powder in each adjacent thin strip, so that the combination between the thin strips is ideal.

**Figure 10:**
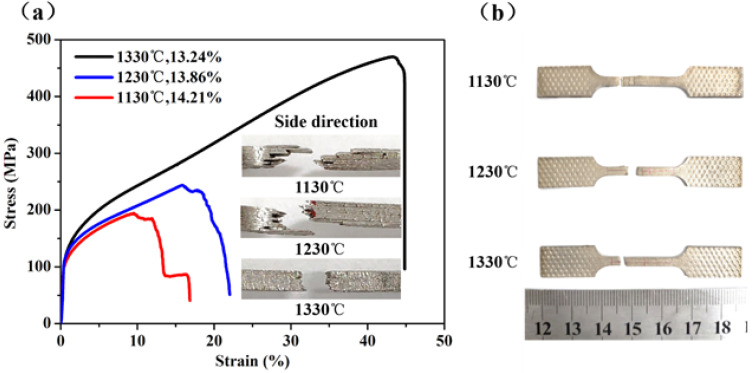
(**a**) The tensile stress-strain curves for the samples sintered at different temperatures; (**b**) Tensile samples after fracture.

The tensile stress-strain curves of the three samples are shown in Figure 10a, the higher the sintering temperature, the higher were the tensile strengths and plasticity measurements of the samples. When the sintering temperature was 1130 °C, the ultimate tensile strength of the sample (No. S7) was 116MPa and the elongation at total failure was 14%. However, when the sintering temperature was 1230 °C, the ultimate tensile strength of the sample (No. S8) was 175 MPa and the elongation at total failure was 17.5%. By comparison, when the sintering temperature increased by 100 °C, the ultimate tensile strength increased by 60 MPa, and the elongation at total failure increased by 3.5%. When the sintering temperature was 1330 °C, the ultimate tensile strength of the sample (No. S3) was 258 MPa, and the elongation at total failure was 26%. Compared with results of the sample produced at 1230 °C, the ultimate tensile strength increased by 83 MPa and the elongation at total failure was 8.5% higher.

In Figure 10a,b, the macroscopic morphology of the tensile fractures at different temperatures are presented beside the curve, and the macroscopic morphologies of fractures at each temperature are verified and consistent with the corresponding curve. The emergence of a steeply sloped curve for the sample with a sintering temperature of 1130 °C was caused by the layered failure. The curve of the sample with a sintering temperature of 1230 °C shows a slow fracture in the fracture stage, and the macroscopic fracture morphology of the sample produced at 1230 °C also shows that the delamination fracture was not very serious. When the sintering temperature was 1330 °C, the fracture of the sample occurred almost instantaneously because the metallurgical bonding was good and there was no phenomenon of layered failure, as shown in the macroscopic fracture morphology of the Figure 10b.

The change of tensile properties seen for sintering temperatures from 1230 to 1330 °C was more apparent than that from 1130 to 1230 °C. After sintering at a temperature of 1130 °C, the quality of combination between sintering thin strips was poor, the delamination was distinct between thin strips due to the action of tensile force. The sample continued to be affected by tension after delamination; the fracture started from one of the thin strips, then spread to the other thin strips in sequence, leading to poor mechanical properties. When the sintering temperature increased to 1230 °C, the material migration was more obvious, which made the sintering neck grow and thicken, and the combination between the thin strips became stronger. The higher the temperature, the bigger the diffusion coefficient of the atom. That means that the probability of metallurgical bonding between adjacent thin strips increases greatly. The thin strips form the joint due to metallurgic combination, so more tensile force is needed to break the sample. At the sintering temperature of 1330 °C, the sintering neck grew and thickened more obviously. Each adjacent thin strip had metallurgical bonding and sintered together to form a whole. Because of the good combination, the strength and plasticity improved significantly.

The tensile curves of the samples with sintering temperatures of 1130 °C and 1230 °C have more similarities, and four stages of deformation are visible. With the wire mesh as the reinforcement, the crack of the material occurred step by step along the metallurgical defect, the stress was redistributed after the fracture, and then the stress decreased gradually, the crack expanded slowly, and the sample finally fractured, so the curves show the shape of a slow and unstable gradient at the fracture stage. However, the sample with sintering temperature of 1330 °C fractured rapidly and completely after the appearance of the crack, which is similar to the fracture characteristics of a compact body.

**Figure 11:**
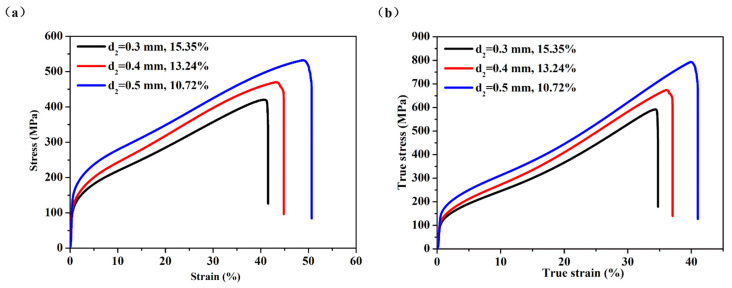
(**a**) The engineering tensile stress-strain curves for the samples with different gaps of the powder box; (**b**) The true tensile stress-strain curves for the samples with different gaps of the powder box.

Three samples (No. S1, S3, S4) were fabricated with the following technological parameters: d_1_ was 0 mm, the number of layers by folding was 10, and the sintering temperature was 1330 °C, d_2_ was 0.3 mm, 0.4 mm, or 0.5 mm, respectively. The porosities for the three samples were 15.35%, 13.24%, and 10.72%, respectively. As shown in Figure 11a, it is clear that, with the reduction of the gap of the powder box, the porosity of the sample increased, and the mechanical properties of the sample decreased. When sample with porosity was 10.72%, the ultimate tensile strength was 532 MPa and the elongation at total failure was 51.62%, and when sample with porosity was 15.35%, the ultimate tensile strength was 420 MPa and the elongation at total failure was 41.52%. When porosity increased by 4.63%, ultimate tensile strength decreased by 21.05%, and the elongation at total failure decreased by 20.09%. Figure 11b is the true tensile stress-strain curves for the samples with different gaps of the powder box, which is derived from Figure 11a through engineering stress-strain and true stress-strain formulas. It can be seen from Figure 11b that the change rule of the curve is consistent with that of Figure 11a. Compared with the sample with porosity of 10.72%, the ultimate tensile strength of the sample with porosity of 15.35% decreased by 25.34%, and the elongation at total failure decreased by 15.29%.

With the increase of the gap of the powder box, the powder flow out from powder box due to the movement of the wire mesh increased, so the layer of powder thickened. Under the same area, more powder was rolled more compactly. The combination of such a large and dense powder with wire mesh, reduced the number of pores and pore size, resulting in a decrease in porosity after sintering. In the same area, if the thickness of powder was different, the mechanical properties were different after sintering. The more powder, the more metallurgical bonding was produced between powder and wire mesh through high temperature sintering, thus showing stronger mechanical properties.

**Figure 12:**
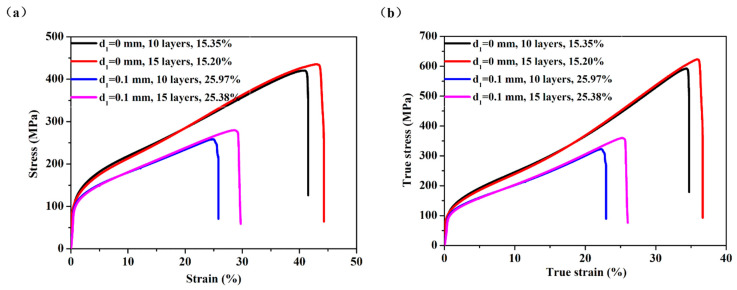
(**a**) The engineering tensile stress-strain curves for the four samples; (**b**) The true tensile stress-strain curves for the four samples.

Two samples (No. S1, S2) were fabricated with the following technological parameters: d_1_ was 0 mm, d_2_ was 0.3 mm, the sintering temperature was 1330 °C, and the number of layers by folding were 10 or 15, respectively. Two other samples (No. S5, S6) were fabricated with the following technological parameters: d_1_ was 0.1 mm, d_2_ was 0.3 mm, the sintering temperature was 1330 °C, and the number of layers by folding were 10 or 15, respectively. The porosities for the four samples S1, S2, S5, and S6 were 15.35%, 15.20%, 25.97%, and 25.38% respectively. In Figure 12a, it can be seen that with the same gap of the roller and gap of the powder box, the porosity of the samples with the number of layers by folding of 10 and 15 were similar. This is because the porosity of the sample is determined by the porosity of the thin strip, and errors are unavoidable in the process of preparation and measurement. Although the number of layers by folding is different, the tensile curve at the stages of the linear elastic deformation, elastic and plastic deformation, and plastic deformation are almost coincident, and the fracture stage is short. Compared with the two samples with 10 layers by folding (No. S1, S5), the ultimate tensile strength of the samples with 15 layers (No. S2, S6) increased by 3.57% and 8.95%, and the elongation at total failure increased by 6.67% and 14.80%, respectively. Figure 12b is the true tensile stress-strain curves for the four samples, which is derived from Figure 12a shows the engineering stress-strain and true stress-strain formulas. It can be seen from Figure 12b that the change rule of the curve is consistent with that of Figure 12a. Compared with the two samples with 10 layers (No. S1, S5), the ultimate tensile strength of the samples with 15 layers (No. S2, S6) increased by 5.06% and 12.18%, respectively, and the elongation at total failure increased by 5.46% and 12.28%, respectively. This is because the samples with 15 layers have more wire mesh as reinforcement to resist deformation and fracture due to tension than do the samples with 10 layers.

As shown in Table 2, by comparing the mechanical properties of the samples with different gaps of the rollers, the rolling force is different with different gaps. The porosity of the sample with d_1_ of 0.1 mm is 25.9%, which is significantly higher than that of 15.35% with d_1_ of 0 mm. The rolling force with d_1_ of 0.1 mm is less than d_1_ of 0 mm, resulting in different densities of powder after rolling, different porosities of the samples, and different mechanical properties.

## 4. Conclusions

The porous, thin strip was fabricated by the composite rolling of stainless steel wire mesh and stainless steel powder. The porous plates of stainless steel wire mesh-powder composites (SWMPCs) with porosities of 10–30% were prepared by folding, pressing, and vacuum sintering with the thin strip. The combination of the SWMPCs have a certain stiffness is well.

The following are the primary conclusions of the present study:

- The permeability of the SWMPCs increased with increasing porosity. As the flowing rate increased, the variation range of pressure differences with high porosity was smaller than that with low porosity. The maximum relative permeability coefficient of the sample with porosity of 25.97% was 51.98% higher than that of the sample with porosity of 10.72%.
- The tensile test results showed that the sintering temperature had a great influence on the tensile properties of the SWMPCs: The mechanical properties of the SWMPCs were enhanced with increased sintering temperature. The whole bonding quality of the sample sintered at 1330 °C was far better than that of the sample at 1130 °C.
- The gap of the roller had a direct effect on the porosity of the SWMPCs, thereby affecting on the tensile properties specifically: The greater the gap of the roller, the higher the porosity and the worse the mechanical properties. The rolling force with d_1_ of 0.1 mm was less than that with d_1_ of 0 mm. As the gap of the roller increased from 0 mm to 0.1 mm, the porosity increased by 69.18%, the ultimate tensile strength decreased by 38.81%, and the elongation at total failure decreased by 37.86%.
- The gap of the powder box also had an important effect on the porosity and tensile properties of the SWMPCs. As the gap of the powder box increased, the porosity decreased and the tensile properties improved. Compared with the samples numbered S1 and S4, when the gap of powder box increased from 0.3 to 0.5 mm, the porosity decreased by 30.16%, the strength increased by 26.67%, and the total elongation of the fracture increased by 24.33%.
- The number of layers by folding had no effect on the porosity of the porous plate, because the porosity of the sample was determined by the porosity of the thin strip. However, the more layers there were, the better were the mechanical properties of the samples. This was because more wire mesh acted as a reinforcement to resist deformation and fracture due to tension.

## Figures and Tables

**Table 1:** The parameters of the permeability of the samples.

Porosity	Test Area (cm^−2^)	Gas Flow (L·min^−1^)	Pressure Difference (kPa)	Relative Permeability Coefficient (m^3^·(h·kPa·m^2^)^−1^)
10.72%	18.10	1.67	24.00	9.22
		3.33	38.00	11.64
		5.00	61.00	10.88
15.35%	18.10	3.33	31.00	14.27
		5.00	40.00	16.59
		6.67	60.00	14.74
25.97%	18.10	5.00	38.00	17.46
		6.67	50.00	17.69
		8.33	65.00	17.01

**Table 2:** Summary of the tensile properties for samples of the stainless steel wire mesh-powder composites (SWMPCs).

Sample Number	d_1_ (mm)	d_2_ (mm)	Number of Layers	Sintering Parameters	Porosity after Sintering (%)	Ultimate Tensile Strength (MPa)		Elongation at Total Failure (%)	
						Engineering	True	Engineering	True
S1	0	0.3	10	1330 °C × 2 h	15.35	420	592	41.52	34.74
S2	0	0.3	15	1330 °C × 2 h	15.20	435	622	44.29	36.64
S3	0	0.4	10	1330 °C × 2 h	13.24	470	673	44.83	37.05
S4	0	0.5	10	1330 °C × 2 h	10.72	532	793	51.62	41.01
S5	0.1	0.3	10	1330 °C × 2 h	25.97	257	320	25.80	22.96
S6	0.1	0.3	15	1330 °C × 2 h	25.38	280	359	29.62	25.78
S7	0.1	0.3	10	1130 °C × 2 h	14.21	194	/	16.82	/
S8	0.1	0.3	10	1230 °C × 2 h	13.86	243	/	21.96	/

## Data Availability

The data presented in this study are available from the corresponding author upon a reasonable request.
